# Levofloxacin versus placebo for the prevention of tuberculosis disease in child contacts of multidrug-resistant tuberculosis: study protocol for a phase III cluster randomised controlled trial (TB-CHAMP)

**DOI:** 10.1186/s13063-018-3070-0

**Published:** 2018-12-20

**Authors:** James A. Seddon, Anthony J. Garcia-Prats, Susan E. Purchase, Muhammad Osman, Anne-Marie Demers, Graeme Hoddinott, Angela M. Crook, Ellen Owen-Powell, Margaret J. Thomason, Anna Turkova, Diana M. Gibb, Lee Fairlie, Neil Martinson, H. Simon Schaaf, Anneke C. Hesseling

**Affiliations:** 1 Centre for International Child Health, Department of Paediatrics, Imperial College London, Norfolk Place, London, W2 1PG UK; 2 Desmond Tutu TB Centre, Department of Paediatrics and Child Health, Faculty of Medicine and Health Sciences, Stellenbosch University, Clinical Building, Room 0085, PO Box 19063, Tygerberg, South Africa; 3 Institute of Clinical Trials and Methodology, MRC Clinical Trials Unit at University College London, London, UK; 4 Wits Reproductive Health & HIV Institute, University of the Witwatersrand, Johannesburg, South Africa; 5 Perinatal HIV Research Unit, University of Witwatersrand, Johannesburg, South Africa

**Keywords:** Tuberculosis, TB, Resistant, Trial, Prevention, Randomised, MDR, Placebo, Child

## Abstract

**Background:**

Multidrug-resistant (MDR) tuberculosis (TB) presents a challenge for global TB control. Treating individuals with MDR-TB infection to prevent progression to disease could be an effective public health strategy. Young children are at high risk of developing TB disease following infection and are commonly infected by an adult in their household. Identifying young children with household exposure to MDR-TB and providing them with MDR-TB preventive therapy could reduce the risk of disease progression. To date, no trials of MDR-TB preventive therapy have been completed and World Health Organization guidelines suggest close observation with no active treatment.

**Methods:**

The tuberculosis child multidrug-resistant preventive therapy (TB-CHAMP) trial is a phase III cluster randomised placebo-controlled trial to assess the efficacy of levofloxacin in young child contacts of MDR-TB cases. The trial is taking place at three sites in South Africa where adults with MDR-TB are identified. If a child aged < 5 years lives in their household, we assess the adult index case, screen all household members for TB disease and evaluate any child aged < 5 years for trial eligibility. Eligible children are randomised by household to receive daily levofloxacin (15–20 mg/kg) or matching placebo for six months. Children are closely monitored for disease development, drug tolerability and adverse events. The primary endpoint is incident TB disease or TB death by one year after recruitment. We will enrol 1556 children from approximately 778 households with an average of two eligible children per household. Recruitment will run for 18–24 months with all children followed for 18 months after treatment. Qualitative and health economic evaluations are embedded in the trial.

**Discussion:**

If the TB-CHAMP trial demonstrates that levofloxacin is effective in preventing TB disease in young children who have been exposed to MDR-TB and that it is safe, well tolerated, acceptable and cost-effective, we would expect that that this intervention would rapidly transfer into policy.

**Trial registration:**

ISRCTN Registry, ISRCTN92634082. Registered on 31 March 2016.

**Electronic supplementary material:**

The online version of this article (10.1186/s13063-018-3070-0) contains supplementary material, which is available to authorized users.

## Background

Tuberculosis (TB) infection signifies that *Mycobacterium tuberculosis* (*M. tuberculosis*) is present in the body without causing symptoms, signs or radiological changes. TB disease, alternatively, is associated with symptoms, signs or radiological changes. Exposure to a person with TB disease can lead to infection with *M. tuberculosis*. Providing preventive therapy to exposed contacts is a viable strategy for reducing their risk of developing TB disease. This is especially so for young children and people living with HIV who, in the absence of appropriate preventive therapy, have a higher risk of disease progression following exposure to and infection with *M. tuberculosis* [1, 2].

The World Health Organization (WHO) estimated that 558,000 individuals developed multidrug-resistant (MDR) TB disease in globally in 2017 [3]. MDR-TB is defined as TB disease caused by *M. tuberculosis* resistant to rifampicin and isoniazid. With the rollout of rapid molecular diagnostic tools including Xpert MTB/RIF, the number of diagnosed adult MDR-TB cases is increasing, with associated increasing numbers of child contacts identified. Modelling suggests that two million children are currently living with MDR-TB infection [4] and of the children infected with *M. tuberculosis* who progress to disease, 90% will do so within 12 months [5]. While treatment outcomes for MDR-TB disease in children are considerably better than in adults [6, 7] the treatment is complex, long, poorly tolerated and associated with frequent and significant adverse events (AE), including ototoxicity, thyroid dysfunction, nausea and vomiting [6, 8–10]. Child-friendly formulations of second-line TB drugs are limited and MDR-TB disease is expensive to treat [11, 12] with prolonged hospitalisation being common for children. Prevention of MDR-TB disease in children is therefore of paramount importance. The United States Centers for Disease Control identified the need for a trial of MDR-TB preventive therapy in 1992 [13]. Since then numerous international agencies have also recommended that such a trial should be a global health priority [14–20] but none have been completed to date, despite the global increase in MDR-TB.

There is strong evidence for the efficacy of isoniazid to reduce the risk of progression to TB disease in child and adult contacts of people with drug-susceptible TB [21, 22]. Isoniazid monotherapy, given daily for six months, is therefore recommended by the WHO in children aged < 5 years and HIV-infected individuals, regardless of age, following exposure to infectious drug-susceptible TB [5, 21–24]. However, the WHO does not currently recommend any specific drug regimen for the contacts of people who are living with infectious MDR-TB. No randomised controlled trials have been completed to assess the efficacy of any regimen to prevent MDR-TB [25], but a recent systematic review and meta-analysis of multiple observational studies of MDR-TB preventive therapy concluded that although the results should be interpreted cautiously, a number of different regimens were associated with a reduction in the risk of subsequent TB disease and were cost-effective [26]. Our primary hypothesis is that levofloxacin, given daily for six months, will protect children exposed to MDR-TB from developing TB disease. We also hypothesise that this treatment will be well tolerated, safe and cost-effective.

## Methods

### Trial design

The tuberculosis child multidrug-resistant preventive therapy (TB-CHAMP) trial is a phase III cluster randomised placebo-controlled trial to assess the efficacy of levofloxacin to prevent TB disease in young (aged < 5 years) child contacts of people with MDR-TB disease.

### Rationale for trial design

#### Target population

This trial specifically targets child household contacts of MDR-TB cases aged < 5 years for two reasons. First, children aged < five years are at the highest risk of progressing to TB disease following infection [2]. Second, concordance of drug susceptibility test (DST) results is high between adults with MDR-TB and young child household contacts [27–29]. Young child household contacts are therefore most likely to benefit from MDR-TB preventive therapy. In addition, global policy and most national guidelines in high-burden settings recommend TB preventive therapy only for child contacts aged < 5 years or for children living with HIV, following exposure to a person with drug-susceptible TB [30]. Although children aged > 5 years, living with HIV, would likely benefit from preventive therapy, the potential for reduced concordance with the identified source case meant that the decision was made to restrict the trial to children aged < 5 years. Children aged < 5 years will be enrolled, regardless of tuberculin skin test (TST) or interferon-gamma release assay (IGRA) status. This is a deliberate decision to ensure programmatic relevance, since the WHO and most National TB Programmes in high-burden TB settings do not require a positive test of TB infection before initiating preventive therapy in children who have been exposed to a person living with infectious drug-susceptible TB [31].

#### Choice of intervention regimen

We considered a wide range of anti-TB drugs and drug combinations for the intervention arm of the trial, including first-line TB drugs, third generation fluoroquinolones, ethionamide, *para*-aminosalicylic acid (PAS), cycloserine, linezolid and clofazimine, and the novel drugs, bedaquiline and delamanid. We discounted drugs if: (1) resistance was likely to be present to that drug in MDR strains (the first-line drugs) [32, 33]; (2) the drug is likely to be poorly tolerated (ethionamide and PAS); (3) the drug is associated with frequent or serious AEs (cycloserine, linezolid, clofazimine); (4) the drug is only weakly effective in killing *M. tuberculosis* (cycloserine, clofazimine, PAS); or (5) appropriate formulations are unavailable or pharmacokinetic parameters are not well understood (delamanid and bedaquiline).

We therefore decided to use a third-generation fluoroquinolone. These drugs have good efficacy against *M. tuberculosis* in vitro and are a core component of MDR-TB disease treatment regimens in both adults and children. Historically, clinicians had been hesitant to use fluoroquinolones in children following early animal research demonstrating cartilage damage in juvenile beagles [34]. However, a significant body of evidence has now demonstrated that drugs of this class are safe in children, even for long-term use [9, 35–38]. Although moxifloxacin has good efficacy against metabolically active mycobacteria, levofloxacin may have better activity against metabolically inactive mycobacteria [39]. A mouse model which evaluated a number of novel TB infection treatment regimens in a latent infection model found that levofloxacin, moxifloxacin and isoniazid had similar efficacy against drug-susceptible strains of *M. tuberculosis.* Moxifloxacin is poorly tolerated in its current formulation due to its bitter taste and there are challenges in taste masking the active ingredient [40]. In addition, the large milligram size of the tablet (400 mg) makes dosing challenging in children. Levofloxacin, in contrast, has a low tablet milligram dose, is licensed for children, has been widely used to treat paediatric MDR-TB disease and other bacterial infections, has known pharmacokinetic parameters when used at once-daily dosing [41], does not interact with antiretroviral therapy drugs and appears to have low toxicity at currently used dosages [42].

Some limited rationale exists for the additional use of isoniazid in a preventive therapy regimen, as it would be efficacious in preventing disease if the child had been exposed to a person with drug-susceptible TB in addition to the identified MDR-TB case. As young children are likely to be highly concordant with the identified source case, we anticipate few children acquiring drug-susceptible organisms. Isoniazid may also provide some efficacy against MDR strains with *inhA* promoter region mutations (typically conferring low-level resistance) [43]. Globally, however, isoniazid resistance is more commonly caused by *katG* gene mutations [44], in which isoniazid is less likely to be effective. For these reasons we elected not to include isoniazid in the intervention regimen. Paediatric pharmacokinetic and safety data for delamanid are becoming increasingly available and the WHO now recommends delamanid for the treatment of MDR-TB disease in children aged as young as six years, but not yet to the youngest children, where treatment of infection is most needed [45]. We plan to adapt the trial design to include delamanid to treat child contacts of fluoroquinolone-resistant MDR-TB, in an observational cohort, once pharmacokinetic data and formulations become available for the youngest children, dependent on additional funding.

In designing our intervention, we also considered that a simple regimen with one drug would likely be safer and would facilitate easier adherence for children. Although concerns have been raised that the use of a single drug could lead to the acquisition of secondary resistance in individuals who develop TB disease, this has not been shown to occur with the use of isoniazid as a single agent for TB preventive therapy in adult and child contacts of drug-susceptible TB [21, 46]. We therefore decided to use levofloxacin alone.

#### Choice of control regimen

As outlined above, isoniazid may be active against *M. tuberculosis* with *inhA* promoter region mutations, as well as susceptible strains from exposures other than the identified index case. For these reasons, the PHOENIx randomised controlled trial (A5300/IMPAACT2003), evaluating MDR-TB infection treatment with delamanid, chose isoniazid for their control arm. However, as also stated above, we anticipate high concordance between index case and child contact (with few children infected with drug-susceptible strains), as well as globally limited utility of isoniazid to treat MDR strains (due to the preponderance of *katG* gene mutations worldwide). In addition, the inclusion of isoniazid would increase the (albeit low) risk of harm that could arise through the use of a potentially hepatotoxic drug. Given the lack of evidence for the efficacy for isoniazid to treat MDR-TB infection and the lack of guidance on this topic, the protocol team, after broad scientific and ethical consideration, determined that it would be most appropriate to use no active drug in the control arm. This represents current WHO guidance [31].

#### Blinding

We considered using an open-label design. However, the primary trial endpoint, incident TB, although standardised to as great a degree as possible, remains, to some extent, subjective. Many children who develop incident TB during the trial will be diagnosed clinically (i.e. not bacteriologically confirmed). The study team felt that with an open-label design there may be bias in the ascertainment of the endpoint. Children known not to be receiving any medication may be followed more closely and investigated more intensively than children known to be on treatment. In addition, there would be potential for bias in the ascertainment and reporting of AEs, with families/caregivers potentially more likely to report AEs if they knew their child was taking an active treatment. We therefore decided blinding and use of placebo would support unbiased assessment of endpoints and toxicity.

#### Clustering

As we planned to identify and treat children in their households, we felt that all children in the same household should be randomised to the same trial arm. Randomisation by household allows for ease of drug administration and protection from bias through accidental or deliberate switching of tablets (levofloxacin/placebo) between child household contacts. The statistical implications of the clustering are accounted for in the sample size estimation and analysis plan.

### Context

South Africa had an estimated TB incidence of 834 cases per 100,000 in 2015 with 3.5% of new cases and 7.1% of previously treated TB cases having either MDR-TB or rifampicin-resistant-TB [3]. The national HIV antenatal prevalence was 29.7% in 2013 [47]; in 2015, of all TB patients with known HIV status, 57% were estimated to be HIV-infected [3]. The trial is being conducted at three sites in South Africa, all of which have extensive research experience in TB trials: (1) Desmond Tutu TB Centre, Stellenbosch University, Cape Town; (2) Perinatal HIV Research Unit, Wits Health Consortium, Klerksdorp; and (3) Wits Reproductive Health and HIV Institute, Wits RHI Shandukani Research Centre, Johannesburg. Trial management, statistical design and analysis, and clinical event management is provided by the Medical Research Council Clinical Trials Unit (MRC CTU) at University College London. The trial began recruitment in September 2017 and recruitment is planned over 18–24 months. All children receive 24 weeks of therapy and are followed for 72 weeks after treatment.

### Trial endpoints

The primary endpoint is incident TB disease (bacteriologically confirmed or clinically diagnosed) or TB death by 48 weeks following randomisation. Incident TB and cause of death are adjudicated by an independent Endpoint Review Committee blinded to treatment allocation, based on available clinical, radiological, microbiological and molecular data using standard case definitions (see Table 1). Secondary endpoints are: (1) all-cause mortality; (b) AEs ≥ grade 3 (possibly or likely associated with drug treatment) during 24 weeks of treatment; (3) percentage of levofloxacin or levofloxacin-placebo doses ingested and retained over 24 weeks of treatment; (4) TB disease over 96 weeks; and (5) incidence of levofloxacin-resistant TB disease.

**Table 1:** Clinical and radiological/laboratory criteria required to make a diagnosis of confirmed, probable or possible TB in child TB contacts aged < 5 years of age. Developed in collaboration with the trial teams from V-QUIN and PHOENIx

Clinical (A)	Radiological/laboratory (B)
• Cough or cervical neck mass (≥2 × 2 cm) for > 2 weeks despite a course of antibiotics • Fever or lethargy for > 1 week despite a course of antibiotics • Documented failure to thrive, i.e. flattening of weight curve crossing centiles, documented weight loss, e.g. > 5%, moderate or severe malnutrition (Weight-for-height Z score < − 2) in relation to previous measures • Classic gibbus suggestive of spinal TB • Depressed level of consciousness, new onset seizures or focal neurological signs suggestive of TB meningitis	• AFBs or caseating granulomas on microscopy (not confirmed by culture or Xpert to be TB) • CXR suggestive of TB (concurrence between two blinded CXR reviewers, with conflicts resolved by third reviewer) despite a course of antibiotics • CSF suggestive of TB (white cell count 10-500 cells per µl with a lymphocyte predominance, protein > 1 g/dL, glucose < 2.2 mmol/L) • Pleural aspirate or ascitic tap with WBC counts, protein, and glucose levels suggestive of TB, consider ADA • CT brain suggestive of CNS TB

• Confirmed TB: positive *M. tuberculosis* + at least one of either A or B

• Positive *M. tuberculosis:* (adapted from Graham et al. [57]): at least one positive culture (with confirmed *M. tuberculosis* speciation) or one positive WHO-endorsed NAAT (e.g. XpertMTB/RIF assay) from respiratory samples (expectorated/induced sputum or gastric aspirate) or other samples such as fine needle aspiration biopsy or other fluid or tissue samples

• Probable TB: at least one of A and at least one of B

• Possible TB: at least one of A or B (but not both) and a decision to treat

• Not TB: the absence of clinical, radiological or laboratory evidence that meets any of the above criteria

• TB infection: immunological evidence of infection with *M. tuberculosis* (TST/IGRA) plus classification as ‘Not TB’.

• Indeterminate/unclassifiable TB status: documented results of the diagnostic evaluation (suspicious symptoms, chest radiograph, laboratory tests) are insufficient for the Endpoint Review Panel to reach determination

• Death: mortality will be classified as death from any cause. TB deaths will be verified using available data (including death certificate, post mortem, hospital and other clinical information or other as available). The Endpoint Review Committee will review and determine the cause of death, if there are any deaths, in all children participating in the trial. Death occurring during a TB episode will be classified as TB death, unless there is clear evidence that the death is unrelated (e.g. motor vehicle accident)

*TB* tuberculosis, *AFBs* acid-fast bacilli, *CXR* chest radiograph, *ADA* adenosine deaminase, *CNS* central nervous system, *CSF* cerebrospinal fluid, *CT* computed tomography, *IGRA* interferon-gamma release assay, *TST* tuberculin skin test, *WBC* white blood count, *WHO* World Health Organization, *NAAT* nucleic acid amplification test

### Trial conduct

#### Index case identification

**Fig. 1:**
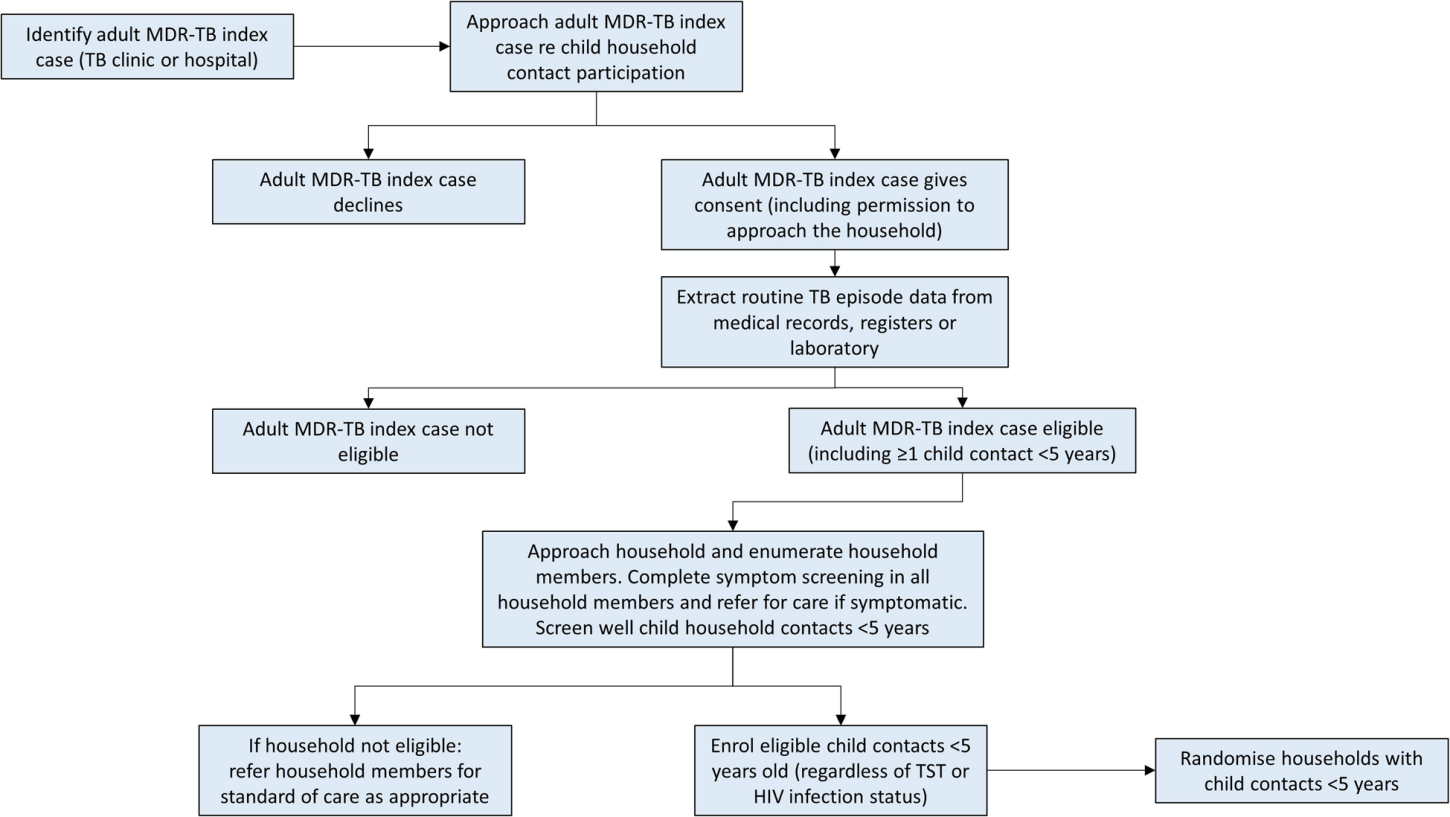
The series of activities conducted by the study team to identify eligible child contacts of multidrug-resistant tuberculosis index cases

Adult MDR-TB index cases are identified via routine National TB Programme TB clinics/hospitals or via other (e.g. laboratory-based) surveillance methods and assessed for eligibility as index cases (Table 2). Adults are approached at their local clinic/hospital or at home to provide consent for collection of their TB episode data and a sputum sample, and for permission to conduct a home visit to enumerate all household members and eligible child contacts (Fig. 1).

**Table 2:** Inclusions and exclusion criteria for adult index cases and child trial participants

Adult index case inclusion criteria	Child participant inclusion criteria	Child participant exclusion criteria
1. Age ≥ 18 years 2. Bacteriologically confirmed pulmonary TB diagnosed from a sputum sample within the preceding 6 months 3. Genotypic and/or phenotypic resistance to isoniazid and rifampicin^a^ 4. Written informed consent to provide routine TB episode data 5. At least 1 child household contact aged < 5 years reported to have been residing in the same household as the adult index case in the previous 6 months	1. Child aged < 5 years who is a household contact of an enrolled adult MDR-TB index case diagnosed during the previous 6 months^b^ 2. Primary residence in the household of the adult MDR-TB index case 3. Consent from the parent or legal guardian for the child for HIV testing^c^ 4. Consent obtained from the parent or legal guardian for the child to be enrolled in the study	1. TB disease at enrolment 2. Currently on isoniazid or a fluoroquinolone^d^ for ≥ 14 days 3. Treated for TB in the previous 12 months 4. Known concurrent exposure to an isoniazid-susceptible (including rifampicin-monoresistant) index case^e^ 5. Children with myasthenia gravis or Guillain–Barré syndrome

^a^If only tested by Xpert MTB/RIF or other approved molecular tests, the index case can be included if rifampicin-resistant, without other confirmed DST. Rates of rifampicin-resistant, isoniazid-susceptible TB are very low in this context [58]. Samples found to be rifampicin-resistant will subsequently be confirmed by other molecular testing and/or by phenotypic DST

^b^Children aged < 5 years who are identified as having been living in the same household as an enrolled adult MDR-TB index case at any point during the preceding 6 months are eligible, if the exposure has lasted > 2 weeks

^c^HIV-infected and uninfected children will be included

^d^Levofloxacin, moxifloxacin, ofloxacin or ciprofloxacin

^e^Any child found to have been in contact with an index case who has isoniazid- or rifampicin-susceptible TB will be referred to the local clinic to access preventive therapy, as per WHO guidelines

#### Household assessment

Following the evaluation of an adult MDR-TB index case, home visits are undertaken and the household contacts enumerated. Comprehensive infection control procedures are followed to protect staff. Screening is conducted in all household contacts to identify those with prevalent TB disease, to rule out additional individuals who may have drug-susceptible-TB and to identify child household contacts who do not have prevalent TB disease and who are eligible for enrolment. Household characteristics and children’s TB exposure are captured using a standard approach [48]. All household members aged ≥ 5 years with suggestive symptoms are referred for TB investigation and TB/HIV care as appropriate.

#### Definition of household

Households are defined using a standard inclusive definition [49]. A household contact is defined as a person who currently lives or lived in the same dwelling unit or plot of land and shares or shared the same housekeeping arrangements as the adult MDR-TB index case, and where there is reported exposure within six months before the index case starting MDR-TB treatment.

#### Screening children for eligibility

Written informed consent from caregivers is obtained to screen for study entry and eligibility (Table 2). Children aged < 5 years who are identified as having been living in the same household as an enrolled adult MDR-TB index case at any point during the preceding six months are eligible, if the exposure has lasted > 2 weeks. Anthropometric measurements are completed, TB symptoms and signs captured, and chest radiographs (CXR), HIV testing and IGRA (QuantiFERON-TB Gold Plus; Qiagen) done. Any children with suggestive symptoms or signs of intrathoracic or extrathoracic TB disease, or with concerning CXR features, are investigated for TB disease (including sampling for bacteriological testing) and are referred for TB care if TB is diagnosed. If any adult is identified with isoniazid- or rifampicin-susceptible TB in the household, then all children in the household become ineligible. Any child found to have been in contact with an index case who has isoniazid- or rifampicin-susceptible TB will be referred to the local clinic to access preventive therapy, as per WHO guidelines. Prevalent TB in child contacts is defined using standard international case definitions for paediatric TB in contact investigation studies (Table 1) which have been harmonised between three international MDR-TB preventive therapy protocols (TB-CHAMP, V-QUIN and the ACTG/IMPAACT PHOENIx trials). CXR findings suggestive of intrathoracic (pulmonary) TB are defined in Table 3.

**Table 3:** Chest radiograph features characteristic (‘typical or highly suggestive chest radiograph findings’ of TB in children aged < 5 years, by disease severity status)

Non-severe disease	Severe disease
• Uncomplicated LN disease - hilar or mediastinal nodes, nodes with unilateral airway narrowing, nodes with single lobe bronchopneumonia, nodes with segmental opacification (< 1 lobe) • Isolated Ghon focus • Simple pleural effusion	• Complicated LN disease (airway compression with hyperinflation or collapse or bilateral airway compression) • Expansile pneumonia (involving ≥ 1 lobe) • Ghon focus with cavitation • Miliary TB • Complicated pleural effusion (alveolar disease with effusion, pneumothorax, loculated pyopneumothorax with air-fluid level), loculated pleural effusion • Adult-type cavitary disease • Bronchopneumonic consolidation with or without cavities or visible lymph nodes • Suspected pericardial effusion (cardiac enlargement)

*LN* lymph node, *TB* tuberculosis

#### Child enrolment

Carers/legal guardians of child household contacts are approached; consent is obtained for screening and, if eligibility is confirmed, subsequent enrolment.

#### Randomisation

All eligible contacts in a household consenting to participate are allocated to the same study arm, but participation of all eligible child household contacts is not required for the household to participate. Enrolment of children and randomisation of households into the study is via a centralised web-based system to maintain allocation concealment. Randomisation is stratified by site and households are randomised 1:1 to either levofloxacin or placebo. The randomisation lists are prepared by an individual who is not involved in the day-to-day running of the trial.

#### Interventions

Medications are dosed by pragmatic weight-bands (Table 4). Children randomised to the intervention arm receive levofloxacin at a target dose of 15–20 mg/kg per day once daily for 24 weeks. Although this is the recommended target dose [50], we acknowledge that higher dosages may be needed to achieve the serum concentrations seen in adults when dosed with 750 mg once daily [51]. In addition, as can be seen in Table 4, the use of weight-bands leads to some over-dosing and some under-dosing for children at the boundaries of each weight-band. Children randomised to the control arm receive placebo formulated to look and taste identical to the 250 mg (or 100 mg) levofloxacin dispersible tablet. Drug (and placebo) is being supplied initially as 250 mg tablets. During the course of the trial, a 100 mg dispersible tablet (and placebo) is expected to become available for use in the lower weight-bands.

**Table 4:** Weight-bands for levofloxacin (100 mg and 250 mg tablets) and resulting drug exposures

Weight-bands (kg)		Tablets of levofloxacin 100 (n)	Tablets of levofloxacin 250 (n)	Range of resulting dosages (mg/kg)			
				Levofloxacin 100		Levofloxacin 250	
				Min	Max	Min	Max
3	4.9	0.5	0.25	10	17	13	21
5	6.9	1	0.5	14	20	18	25
7	9.9	1.5	0.75	15	21	19	27
10	11.9	2	1	17	20	21	25
12	15.9	2.5	1	16	21	16	21
16	19.9	3	1.5	15	19	19	23
20	24.9		1.5			15	19
25	29.9		2			17	20

Target dosage for levofloxacin is in the range of 15–20 mg/kg

Children in the placebo arm will receive the same number of tablets based on their weight-band

#### Follow-up

Children are seen at 4, 8, 12, 16, 24, 36, 48, 72 and 96 weeks for study visits and at any time that caregivers feel that the child may have developed TB disease or they, or the study team, have other concerns.

### Data collection

**Fig. 2:**
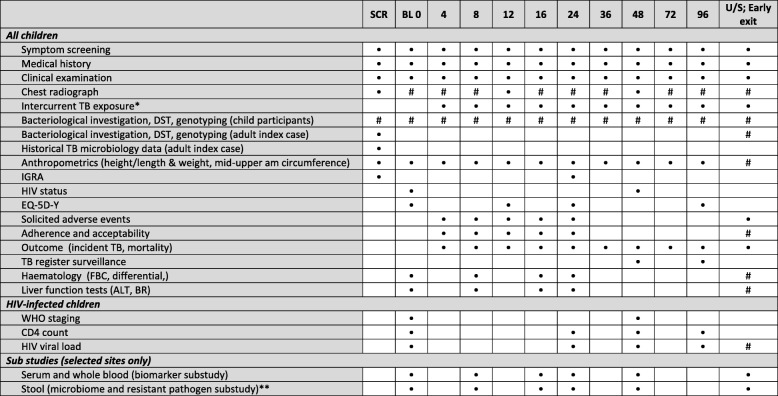
Schedule of evaluations. SCR screening, BL baseline – at randomisation, U/S unscheduled, TB tuberculosis, ALT alanine aminotransferase, BR bilirubin, DST drug susceptibility test, FBC full blood count, IGRA interferon-gamma release assay, WHO World Health Organization. *If intercurrent exposure to an isoniazid- or rifampicin-susceptible TB case, preventive therapy will be offered. **At selected sites only. #Based on clinical indication only – TB symptoms at baseline, or at follow-up: new or persistent symptoms, or CXR changes at any time including endpoint evaluation. HIV testing at 48 weeks will be repeated in HIV-negative participants. If HIV status is already known to be positive at screening, the CD4 count and HIV viral load should be completed at baseline in HIV-infected children. HIV viral load is standard of care in HIV-infected children

The data collected, investigations undertaken and time points at which these are carried out, from the index case, household and child, are indicated in Fig. 2. The TB treatment register at the treating clinic and National Health Laboratory Service database are reviewed by the study team to collect data on the index case. A sputum sample is also collected from the index case for culture, DST and genotyping. At follow-up study visits of the child contacts, anthropometric measurements are recorded and caregivers are asked questions regarding the child. Stool is collected at selected sites for non-mycobacterial microbiology and microbiome analysis. Any child with symptoms or signs suggestive of TB disease, a CXR with features consistent with TB disease, or a CXR with persistent abnormalities despite a course of antibiotics, will be evaluated for TB disease by a clinician and have two respiratory samples (expectorated/induced sputum or gastric aspirate) collected for smear microscopy, GeneXpert MTB/RIF and culture. If the culture is positive, the sample undergoes DST and genotyping.

### Adverse events

Safety assessments are conducted at each visit during the 24 weeks on treatment and include evaluation of symptoms, signs and laboratory investigations. At every study visit (and at unscheduled visits) a symptom checklist prompts for symptoms relating to possible drug toxicities. All symptomatic patients are evaluated by the study doctor and discussed with the site Principal Investigator. Safety laboratory investigations are performed at baseline and at 8, 16 and 24 weeks and are also performed at unscheduled visits if clinically indicated. AEs (clinical and laboratory) are graded using the 2017 Division of AIDS toxicity grading scale [52]. All AEs are recorded in the child’s notes and reported to the MRC CTU and ethics committees as required within agreed timescales. There are no known drug–drug interactions between levofloxacin and antiretroviral drugs used to treat children living with HIV [42] and a recent evaluation of long-term levofloxacin use in children with MDR-TB found no evidence of cardiotoxicity or QTc prolongation [53].

### Statistical considerations

**Fig. 3:**
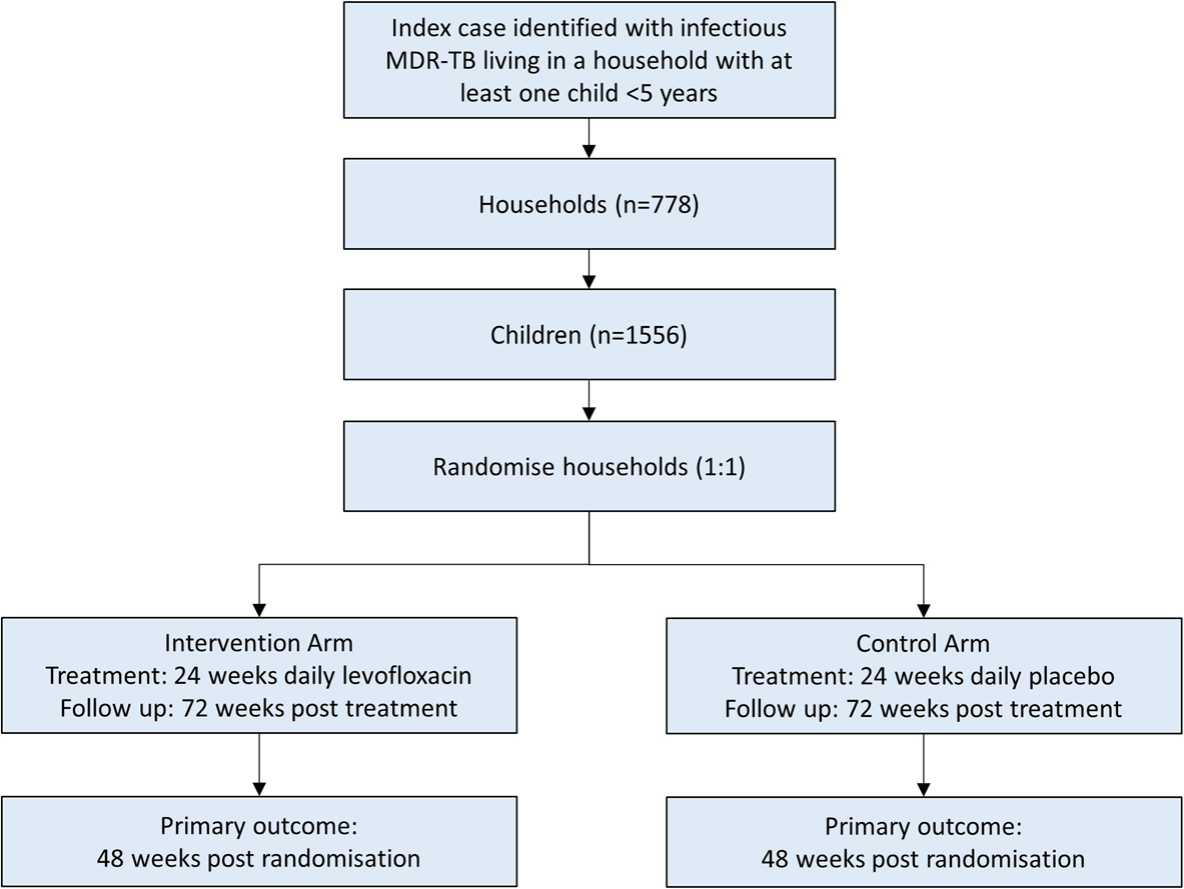
Schema of the trial documenting numbers of households and individuals to be recruited and numbers in each trial arm

For sample size determination, we have assumed a two-sided superiority test (alpha of 0.05), assuming that TB incidence in the control arm is 7% [2]. We have powered the trial to detect a 50% reduction of the TB incidence in the levofloxacin arm. We have conservatively assumed 10% loss to follow-up [21, 35, 54], two children per household [54] and an intra-class correlation within households (a measure of how similar the households are to each other) of 0.1. Other intra-class correlation values and the impact on sample size are shown in Table 5. Considering these assumptions, we will need to enrol 1556 children (778 per arm) to achieve 80% power to detect differences between the two arms (Fig. 3). There will be one formal interim analysis using Haybittle–Peto-type boundaries [55, 56], which will occur after accrual of at least half the targeted number of households or when half the target number of household contacts (whichever is sooner) have been recruited and when these recruited child participants have been followed for at least six months. Primary analysis is intention to treat.

**Table 5:** Total number of households (children)^a^ required for 80% and 90% power for varying control arm event rates and intra-cluster correlation coefficients

Power	Contacts who develop TB disease by 48 weeks (control arm) (%)	Intra-cluster correlation coefficients		
		0.05	0.1	0.15
*80%*	*5*	1056 (2122)	1108 (2216)	1158 (2316)
	*7*	742 (1484)	*778 (1556)*	814 (1628)
	*10*	508 (1016)	532 (1064)	556 (1112)
*90%*	*5*	1414 (2828)	1480 (2960)	1548 (3096)
	*7*	994 (1988)	1040 (2080)	1088 (2174)
	*10*	678 (1356)	710 (1420)	742 (1484)

^a^Assumes 2 contacts per household, 10% loss to follow-up, two-sided 5% significance level test and a 50% risk reduction in the intervention arm

The number in italics indicates the chosen trial sample size

### Ethical considerations

#### Consent

Written informed consent is obtained from the adult MDR-TB index case to approach the household and the parent/legal guardian of the eligible child for their screening and possible subsequent enrolment into the trial. The consent process is conducted in the home language of the person from whom consent is requested and that individual is given a written information sheet, also in their home language, explaining the study. Consent is obtained before any trial-related procedures are performed.

#### Registration and ethical approval

The trial has been registered, and details can be viewed at: http://www.isrctn.com/ISRCTN92634082. The trial has been approved by the Stellenbosch University Human Research Ethics Committee (M16/02/009), the University of the Witwatersrand Ethics Committee (160409) and the Medicines Control Council, South Africa.

#### Oversight

The trial is run primarily by the Trial Management Group and is overseen by the Trial Steering Committee. An Independent Data Monitoring Committee sees confidential, unblinded data for the trial and advises the Trial Steering Committee on whether the trial needs to be prematurely closed. An Endpoint Review Committee, blinded to treatment arm, evaluates clinical trial endpoints and causes of death.

### Sub-studies

#### Social science

The Social Science team will describe: (1) the perceptions that patients, families and health workers have about TB care and prevention, as well as the TB-CHAMP trial; (2) how the study intervention is implemented in the context of local health systems; and (3) the families’ experiences of the trial. This involves key informant interviews with health service personnel, mixed-methods research around implementation and a nested qualitative cohort.

#### Health economic research

Costing and cost-effectiveness of the trial interventions for both families and health systems at the three collaborating sites will be evaluated. This involves collecting data on costs associated with the interventions as well as costs associated with children developing MDR-TB disease. The primary outcome is the incremental cost-effectiveness ratio of levofloxacin against placebo.

#### Drug studies

Palatability and acceptability of the new child-friendly dispersible 100 mg levofloxacin formulation has been evaluated in 24 children before trial start-up as part of a lead-in pharmacokinetic sub-study. Bioavailability is also being evaluated in healthy adult volunteers. Ongoing evaluation of the 250 mg formulation (and 100 mg formulation when it becomes available) will take place throughout the trial using questionnaires and qualitative research.

#### Other sub-studies

The trial provides a unique platform to conduct a number of basic science studies. Given that children in the placebo arm are monitored closely without treatment (a situation that would not be possible following exposure to drug-susceptible TB, given the proven efficacy of isoniazid preventive therapy), this trial permits evaluation of correlates of risk in MDR-TB-exposed children, using RNA transcriptomic approaches. Blood samples are taken at the time of routine, trial-related blood draws and stored for subsequent analysis. Baseline and serial stool samples are taken from children at selected sites to evaluate the impact of levofloxacin on the microbiome of children and its effect on the development of levofloxacin resistance in non-mycobacterial bacteria. The trial provides an opportunity to use whole genome sequencing to study the molecular epidemiology of *M. tuberculosis*, the impact of drug resistance on transmission and to evaluate concordance between index cases and household contacts.

## Discussion

Should TB-CHAMP determine that levofloxacin is effective in preventing the development of TB disease in young children who have been exposed to MDR-TB, and that it is safe and well tolerated, we would expect that this intervention would rapidly translate into policy. We will continue to inform the WHO and other policy groups about the trial status and findings. Although the study is being conducted in only one country, the diverse nature of the trial sites, with varied host genetics, mycobacterial strain types and varying prevalence of drug resistance, epidemiological characteristics, health systems and cultural practices, would mean that the results are likely to be generalisable to children more broadly.

## Trial status

Recruitment to the trial started in September 2017. Recruitment will continue until the target number of children has been recruited. This is anticipated to take 18–24 months. The current protocol is Version 1.0 (see Additional file 1).

## Additional file

Additional file 1: SPIRIT 2013 Checklist: Recommended items to address in a clinical trial protocol and related documents*. (PDF 129 kb)

## Abbreviations

CXR: Chest radiographs
DST: Drug susceptibility test
IGRA: Interferon-gamma release assay
*M. tuberculosis*: *Mycobacterium tuberculosis*
MDR: Multidrug-resistant
MRC CTU: Medical Research Council Clinical Trials Unit
PAS: *Para*-aminosalicylic acid
TB: Tuberculosis
TB-CHAMP: Tuberculosis child multidrug-resistant preventive therapy trial
TST: Tuberculin skin test
WHO: World Health Organization
